# Inferring cancer progression from Single-Cell Sequencing while allowing mutation losses

**DOI:** 10.1093/bioinformatics/btaa722

**Published:** 2020-08-17

**Authors:** Simone Ciccolella, Camir Ricketts, Mauricio Soto Gomez, Murray Patterson, Dana Silverbush, Paola Bonizzoni, Iman Hajirasouliha, Gianluca Della Vedova

**Affiliations:** Department of Informatics, Systems and Communication, University of Milano-Bicocca, Milan, Italy; Department of Physiology and Biophysics, Tri-I Computational Biology & Medicine Graduate Program, Weill Cornell Medicine of Cornell University, New York, NY 10021, USA; Institute for Computational Biomedicine, Englander Institute for Precision Medicine, The Meyer Cancer Center, Department of Physiology and Biophysics, Weill Cornell Medicine of Cornell University, New York City, NY 10021, USA; Department of Informatics, Systems and Communication, University of Milano-Bicocca, Milan, Italy; Department of Informatics, Systems and Communication, University of Milano-Bicocca, Milan, Italy; Department of Computer Science, College of Arts and Sciences, Georgia State University, Atlanta, GA 30303, USA; Department of Pathology and Center for Cancer Research, Massachusetts General Hospital and Harvard Medical School, Boston, MA 02114, USA; Department of Informatics, Systems and Communication, University of Milano-Bicocca, Milan, Italy; Institute for Computational Biomedicine, Englander Institute for Precision Medicine, The Meyer Cancer Center, Department of Physiology and Biophysics, Weill Cornell Medicine of Cornell University, New York City, NY 10021, USA; Department of Informatics, Systems and Communication, University of Milano-Bicocca, Milan, Italy

## Abstract

**Motivation:**

In recent years, the well-known Infinite Sites Assumption has been a fundamental feature of computational methods devised for reconstructing tumor phylogenies and inferring cancer progressions. However, recent studies leveraging single-cell sequencing (SCS) techniques have shown evidence of the widespread recurrence and, especially, loss of mutations in several tumor samples. While there exist established computational methods that infer phylogenies with mutation losses, there remain some advancements to be made.

**Results:**

We present Simulated Annealing Single-Cell inference (SASC): a new and robust approach based on simulated annealing for the inference of cancer progression from SCS datasets. In particular, we introduce an extension of the model of evolution where mutations are only accumulated, by allowing also a limited amount of mutation loss in the evolutionary history of the tumor: the Dollo-*k* model. We demonstrate that SASC achieves high levels of accuracy when tested on both simulated and real datasets and in comparison with some other available methods.

**Availability and implementation:**

The SASC tool is open source and available at https://github.com/sciccolella/sasc.

**Supplementary information:**

Supplementary data are available at *Bioinformatics* online.

## 1 Introduction

Recent developments in targeted therapies for cancer treatment rely on the accurate inference of the clonal evolution and progression of the disease. As discussed in several recent studies (Morrissy *et al.*, 2016; Wang *et al.*, 2016), understanding the order of accumulation and the prevalence of somatic mutations during cancer progression can help better devise these treatment strategies.

Most of the available techniques for inferring cancer progression rely on data from next-generation bulk sequencing experiments, where only a proportion of observable mutations from a large amount of cells is obtained, without the distinction of the cells that carry them. In recent years, many computational approaches have been developed for the analysis of bulk sequencing data with the purpose of inferring tumoral subclonal decomposition and reconstructing tumor phylogenies (evolutionary trees) (Bonizzoni *et al.*, 2018; El-Kebir *et al.*, 2016; Hajirasouliha *et al.*, 2014; Jiao *et al.*, 2014; Malikic *et al.*, 2015; Marass *et al.*, 2016; Popic *et al.*, 2015; Satas and Raphael, 2017; Strino *et al.*, 2013; Toosi *et al.*, 2019; Yuan *et al.*, 2015). The main drawback of this technique is that a bulk sequencing sample contains a mixture of both healthy and cancerous cells—and this clonal evolution can only be estimated by the proportions of observable mutations.

Single-cell sequencing (SCS) technologies promise to deliver the best resolution for understanding the underlying causes of cancer progression. However, it is still difficult and expensive to perform SCS experiments with a high degree of confidence or robustness. The techniques currently available are producing datasets, which contain a sizeable amount of noise in the form of false negatives from allelic dropout, and missing values due to low coverage. Another issue that these technologies suffer from is the presence of doublet cell captures. However, such issues are slowly fading away and the state-of-the-art in preprocessing steps for removing such artifacts is quite advanced (DePasquale *et al.*, 2019). Hence, we believe that more immediate issues, such as the lack of accuracy reflected in the high dropout and false-negative rates inherent to the technology, call for methods that are able to infer cancer progression from this data produced by current SCS techniques.

Various methods have been recently developed for this purpose (Jahn *et al.*, 2016; Ross and Markowetz, 2016; Zafar *et al.*, 2017, 2019), some of them introducing a hybrid approach of combining both SCS and VAF (bulk sequencing) data (Malikic *et al.*, 2019a; Ramazzotti *et al.*, 2019; Salehi *et al.*, 2017). Most of these methods, however, rely on the Infinite Sites Assumption (ISA), which essentially states that each mutation is acquired at most once in the phylogeny and is never lost. One reason being that such a simplifying assumption leads to a computationally tractable model of evolution, namely, the problem of finding a perfect phylogeny (Gusfield, 1991). This model is safe to use in settings, such as the evolution of natural populations, and tends to be the norm more than the exception in this setting (Kimura, 1969). Cancer progression, however, is a fairly extreme situation, where the evolution is very fast, under attack from the immune system, and with a high mutation rate. As a result, studies of SCS data are beginning to reveal phenomena that cannot always be explained with a perfect phylogeny (Brown *et al.*, 2017; Kuipers *et al.*, 2017). Some articles (Kuipers *et al.*, 2017) reveal widespread recurrence and loss of mutations, while large deletions on several branches of a tree can span a shared locus (Brown *et al.*, 2017), thus, a given mutation may be deleted independently multiple times.

In this work, we propose a novel and more general model to explain the above phenomena, which is not unnecessarily held back by strict adherence to the ISA. Some recent methods are beginning to appear, which have the same objective in mind, such as TRaIT (Ramazzotti *et al.*, 2019), SiFit (Zafar *et al.*, 2017) and SPhyR (El-Kebir, 2018): in detail, TRaIT accounts for violations of the ISA by accommodating for convergent evolution; SiFit accounts for both mutation recurrence and loss without specifying a particular model of evolution; and, on the other hand, SPhyR (independently from our article) utilizes the same phylogeny model used in this work, thus allowing deletions of mutations.

In our approach, we use the Dollo model (Farris, 1977; Rogozin *et al.*, 2006), one of the models that is more general than the perfect phylogeny model, to allow the loss of point mutations. In particular, while the Dollo model still constrains that a mutation can only be *acquired* at most once, it allows any number of independent losses of the mutation. Once we depart from an ideal, error-free, perfect phylogeny model (Gusfield, 1991), we lose its convenient computational tractability: in fact allowing errors or missing data results in an NP-hard problem. Adopting the more general Dollo model is only going to exacerbate the problem. However, if we restrict the number of losses of any mutation to 1 or 2 (rather than strictly 0), the resulting solution space is still small enough to explore a sizable portion of it in a reasonable amount of time, in practice. Moreover, from a biological point of view, one would not expect a mutation to be lost more than a few times, since it is not likely that mutations are widely lost (Brown *et al.*, 2017; Kuipers *et al.*, 2017). Furthermore, all the currently available methods assume that the false-negative rate is the same for all mutations. While this is suitable for samples coming from DNA (i.e. scDNA-seq) data, the false-negative rate of the mutations in samples coming from RNA (i.e. scRNA-seq) data can vary a because of differing levels of gene expression. Since our approach is suitable for both types of data, i.e. a suitable parameter setting can be found for modeling the progression of cancer from samples coming from either DNA or RNA data, to accommodate the latter, our approach also allows a different false-negative rate for each mutation: it is one of the first methods with this feature. In fact, to the best of our knowledge, the only other article with a similar feature has appeared very recently (Wu, 2019); in that article, different false negative and false-positive rates are allowed for each mutation and for each cell. At the same time, mutation losses are not allowed. SciΦ (Singer *et al.*, 2018) also allows different rates, but it is essentially a phylogeny-aware mutation caller, not a tool designed to infer tumor phylogenies.

Here, we introduce the Simulated Annealing Single-Cell inference (SASC) tool, a maximum likelihood phylogeny search framework that allows deletion of mutations, by incorporating the Dollo parsimony model (Farris, 1977; Rogozin *et al.*, 2006). We show that our approach is competitive with the state-of-the art tools for inferring cancer progression from SCS data, while being the only tool to correctly identify important driver mutations in some real datasets, as verified by the manually curated progression scenarios for these data.

## 2 Materials and methods

### 2.1 Formulation of the tree reconstruction problem

As mentioned before, cancer progression reconstruction can be modeled as the construction of a character-based incomplete phylogeny on a set of (cancer) cells, where each character represents a mutation.

In this framework, we consider the input as an *n* ×* m* ternary matrix *I_ij_*, where an entry *I_ij_*=0 indicates that the sequence of cell *i* does not have mutation *j*, *I_ij_*=1 indicates the presence of mutation *j* in the sequence of cell *i*, and a ? indicates that there is not enough information on the presence/absence of mutation *j* in cell *i*. This uncertainty about the presence of a mutation in a cell is a consequence of insufficient coverage in the sequencing, a common scenario in SCS experiments.

However, the uncertainty of some entries is not the only issue that results from the sequencing process. In fact, entries of the input matrix *I* can also contain false positives and false negatives—while the false-positive rate is usually very low, the false-negative rate can be high and can also vary depending on different factors. In particular, for scRNA-seq data, the varying expression levels of different genes can easily lead to different false-negative rates for each mutation, since a highly expressed gene will have significantly higher coverage than an under-expressed gene, resulting in a more accurate single-nucleotide variant (SNV) call for that particular gene. On the other hand, a gene, which is less expressed is likely to have a lower coverage, leading to a less accurate presence/absence estimation in the cells. We assume that these errors occur independently across all the (known) entries of *I*. Namely, if *E_ij_* denotes the final *n* ×* m* output matrix, i.e. the binary matrix without errors and noise estimated by the algorithm, then α_*j*_ denotes the false-negative rate of mutation *j*, and β denotes the false-positive rate, similarly to El-Kebir (2018), Jahn *et al.* (2016), Ross and Markowetz (2016) and Zafar *et al.* (2017). Hence, for each entry of *E_ij_* the following holds:

$$\begin{array}{c} P(I_{ij}=0|E_{ij}=0)=1-\beta \;P(I_{ij}=0|E_{ij}=1)=\alpha_{j}\\ P(I_{ij}=1|E_{ij}=0)=\beta \;P(I_{ij}=1|E_{ij}=1)=1-\alpha_{j}. \end{array}$$

We aim to find a matrix, which maximizes the likelihood of the observed matrix *I* (Jahn *et al.*, 2016) under the probabilities of false positives/negative and missing entries. Differently from previous works, our model also accounts for losses of mutations, thus, we define the prior probability $P(L(j))=\gamma_{j}$—independent from the previous ones—of losing mutation *j* and the set of variables *c_j_* for j=1,…m that denotes the total number of losses for mutation *j* in the evolutionary history. In practice, we expect that a researcher might able to determine that some mutations *j* are very unlikely be lost, therefore setting $\gamma_{j}=0$.

However, we are interested in the reconstruction of the evolutionary history of the input cells, thus the resulting matrix *E* should contain clones (groups of cells with the same mutations) that can be explained by an evolutionary process of the mutations. This restriction motivates the introduction of the concept of phylogenetic tree, or simply phylogeny.

**Fig. 1.**
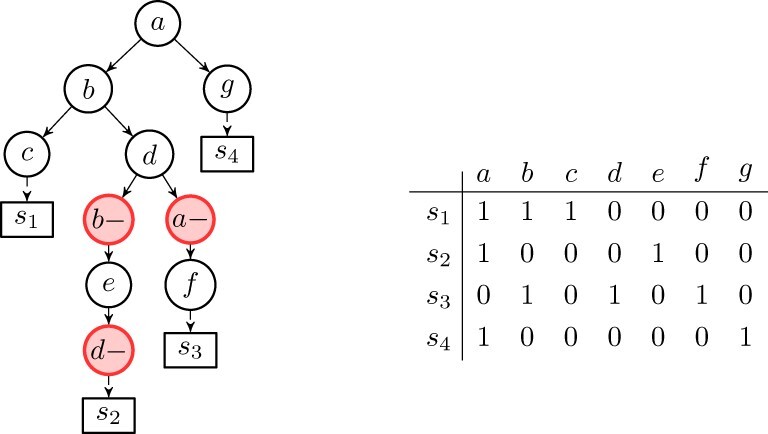
Example of a binary matrix *E* (right) representing a sample of the (*n* = 4) cells $\left\{s_{1}\ldots s_{4}\right\}$ affected by the set C={a…g} of mutations. The tree (left) is a cancer phylogeny *T* explaining this matrix. Note that, the state of the internal node in the tree (left) labeled with (mutation) *f* has state {*b*, *d*, *f*} (mutation *a* appears in the root, but was lost in the path to this node), hence the genotype profile $D(T,s_{3})$ of leaf *s*_3_ in the tree is 0101010. Note that $E_{s_{i}}=D(T,\sigma_{s_{i}})$ holds for the (trivial) mapping $\sigma_{s_{i}}=s_{i}$, hence *T* (left) encodes *E* (right). Informally, leaf *s*_1_ was ‘attached’ to the internal node labeled by *f* because genotype profile $D(T,s_{3})$ of leaf *s*_3_ in *T* matches the row for *s*_3_ in *E*, e.g. Observe that the matrix (right) does not allow a perfect phylogeny, and that the tree (left) is a Dollo-1 phylogeny

A (cancer) phylogeny *T* on a set *C* of *m* mutations and *n* cells (affected by these mutations) is defined as a rooted tree whose internal nodes are labeled by the mutations of *C*, while the leaves are labeled by the cells (see Fig. 1A). Notice that the labeling must satisfy some restrictions depending on the evolutionary model that we consider. For example, in a perfect phylogeny, no two nodes have the same label. This is an alternative, but essentially equivalent, definition of classical character-based phylogeny, where the tree *T* is defined on a set of characters and where leaves have no label and represent different species.

The *state* of a node *x* is defined as the set of mutations that have been acquired but not lost in the path from the root to *x*. The state of each leaf *l* of *T* is naturally represented by a binary vector of length *m*, called genotype profile, that we denote *D*(*T*, *l*), where $D{\left(T,l\right)}_{j}=1$ if and only if the leaf *l* has the mutation *j* and 0 otherwise (see Fig. 1B).

We say that the tree *T encodes* a matrix *E* if there exists a mapping σ of the rows (cells) of *E* to the leaves of *T* such that $E_{i}=D(T,\sigma_{i})$ for each row *i* of *E*, where σ_*i*_ denotes the image of row *i* through the mapping σ. Informally, σ_*i*_ is the node in the phylogenetic tree corresponding to the node where the cell *i* is attached. Notice that the matrix *E* is fully characterized by the pair (T,σ) (see Fig. 1C). Thus, our problem can be expressed as finding the tree *T* that maximizes the following objective function:

$$\max\sum_{j}^{m}[-c_{j}\;\text{log}(1-P(L(j)))+\sum_{i}^{n}\;\text{log}\;(P(I_{ij}|D{\left(T,\sigma_{i}\right)}_{j}))].$$

We point out that the values assigned to the unknown entries of the input matrix do not factor into the objective function, i.e. $P(I_{ij}=?|E_{ij}=1)=P(I_{ij}=?|E_{ij}=0)$. To simplify the computation of the likelihood, we slightly abuse notation in supposing that $P(I_{ij}=?|E_{ij}=1)=P(I_{ij}=?|E_{ij}=0)=1$. Furthermore, σ can be computed directly from *T*; for each tree, we can obtain the best assignment using an exact mapping; leaving *T* as the only variable to optimize.

### 2.2 Introduction of the Dollo-k model

The Dollo parsimony rule assumes that, in a phylogeny, any single mutation is uniquely introduced in the evolutionary history, but deletions of the mutation can occur any number of times. A restricted version of the Dollo model can be obtained by bounding the number of deletions for each mutation. We denote as Dollo-*k* the evolutionary model in which each mutation can be acquired exactly once and can be lost at most *k* times. The special cases, Dollo-0 and Dollo-1, correspond to the perfect (Gusfield, 1991) and persistent (Bonizzoni *et al.*, 2012, 2017; Della Vedova *et al.*, 2017) phylogeny models, respectively. The phylogeny reconstruction problem under a Dollo-*k* model is NP-complete (Goldberg *et al.*, 1996) for any *k* > 1.

Since the Dollo evolutionary model allows back mutations, we introduce a new type of node label in the phylogenetic tree, to express mutational losses. For each mutation *p*, we create *k* new mutations $p_{l}^{-}$ for l∈{1,…,k}, representing the possible losses of mutation *p*. As in the perfect case, we require that no two different nodes have the same label. Additionally, we impose that all nodes labeled by a mutation loss *p*^–^ are descendants of the node labeled by the gain of mutation *p*. Consequently, the vector $D(T,\sigma_{i})$, which expresses the genotype profile of a row *i* will have a 1 in mutations acquired but never lost in the path from the root to the parent σ_*i*_ of the leaf *i*. Note that, the tree of Figure 1D is a Dollo-1 phylogeny. We stress that, unlike the case of the perfect phylogeny, when deletions are introduced, we might have more than one tree that is a solution. For example, switching the labels of nodes *b*^–^ and *d*^–^ in Figure 1 produces a different tree, which is still a solution of the proposed input matrix when the Dollo model is considered. Moreover, the set of ancestral relationships between those two mutations is opposite in both representations. An increase of the number of cells and mutations, coupled with the noise caused by false calls and missing entries, expands the solution space of this problem, increasing the number of different cancer progression phylogenies which equally explain the same input.

#### 2.2.1 Our model

The model, we employ in this work is the Dollo-*k* model, with the added restriction that there are at most *d* total mutation losses in the entire progression. In addition to *k*, this *d* is a user supplied parameter. Note that, with a maximum *d* of total losses in the progression, it means that the variable *c* is subject to (i) $c_{j}\leq k\;\forall j$ and (ii) $\sum_{j}^{m}c_{j}\leq d$. Only a small number of mutation losses in each tumor have been reported (Kuipers *et al.*, 2017), therefore we expect small values of *k* and *d* to be used in practice. Most precisely, we believe that k≤2 and d≤5 in almost all cases. If the number of mutation is not too small, setting d≤5 essentially implies k≤1, hence making the parameter *k* mostly irrelevant. Still, we have decided to keep it because it guarantees that some degenerate trees are never computed. We recall that our model also has the γ_*j*_ parameters, i.e. the prior probability of losing mutation *j*.

### 2.3 Simulated annealing

As mentioned before, the fact that (i) we can flip entries and that (ii), we want to find the maximum likelihood tree, makes the phylogeny reconstruction problem under the Dollo-*k* model computationally hard for any *k* > 0. For this reason, in this article, we consider the Simulated Annealing (Kirkpatrick *et al.*, 1983) (SA) approach in order to find a tree which maximizes the likelihood of an incomplete input matrix and that satisfies the Dollo-*k* phylogeny model, where *k* is given as input.

SA is a random search technique, which explores the region of feasible solutions, searching for an optimal one. As all other meta-heuristic strategies, it is not guaranteed that SA finds the optimal value of the objective function in a finite number of steps; nevertheless, unlike other deterministic search methods, which can be trapped into local optima, SA has been designed to overcome this drawback and converge to a global optimum. The basic idea of the algorithm is to perform a random search, which accepts, with some probability, changes that do not necessarily improve the objective function. At each step, the probability of moving to some state with a smaller value changes according to a parameter called the *temperature*, which continuously decreases as the exploration evolves. In the first iterations of the algorithm execution, the temperature is very high, and it is possible (with a fairly high probability) to accept a move into a state with a lower objective value, but as temperature decreases, the probability of moving also decreases. At the end, when the temperature is sufficiently low, the algorithm becomes a local search method, hence unable to escape a local optimum.

#### 2.3.1 Neighborhood topology

An essential element of a SA approach that we must provide how the algorithm search process can move from a given state to another. In our particular framework, we attempt to find a tree, thus, we must define the neighborhood of a phylogenetic tree in the feasible region, and the algorithm moves from a tree to one of its neighbors. The choice of neighborhood is crucial in the algorithm definition since it determines how feasible solutions are explored, hence, ultimately determining whether or not the algorithm converges.

In our approach, the notion of neighborhood is operational, i.e. two phylogenetic trees are neighbors if one can obtained from the other via some operation from a set we will define shortly. For the sake of clarity, we introduce some notation: given a phylogenetic tree *T* and a node (labeled as) *i*, ρ(i) denotes the parent of *i* in *T*.

- Subtree Prune and Reattach: given a tree *T* and two internal nodes u,v∈T such that neither is an ancestor of the other, we prune the subtree rooted in *u* by removing the edge (u,ρ(u)) and we reattach it as a new child of *v* by adding the edge (v,ρ(v)).
- Add a deletion: given two nodes u,v∈T such that *v* is an ancestor of *u*, we insert a node *v*^–^ that represents a loss of mutation *v*. The new node is made the parent of *u*. We remark that this operation takes place only if the resulting tree satisfies the desired phylogeny model. More precisely, for the Dollo-*k* we must check that the mutation *v* has been previously lost in the tree at most *k* – 1 times, and never lost in any ancestor or descendant of *v*^–^.
- Remove a deletion: given a node u∈T, labeled as a loss, we simply remove it from the tree *T*: all children of *u* are added as children of ρ(u) and the node *u* is then deleted.
- Swap node labels: given two internal nodes u,v∈T, the labels of *u* and *v* are swapped. If a previously added loss becomes invalid due to this operation—because a mutation *c* is lost in a node *c*^–^, but the node where the mutation *c* is acquired is not an ancestor of *c*^–^ anymore—then we remove the deletion *c*^–^.

#### 2.3.2 The algorithm

The goal of the algorithm is to find a maximum likelihood Dollo-*k* phylogeny tree; a SA process is performed using the previously defined set of valid operations according to the same temperature decay process—in each iteration, one of these operations is performed, chosen uniformly among all possible candidate operations. Finally, after a new neighbor is generated, cells are optimally attached to the tree, maximizing its likelihood, resulting in the score of the new solution.

Moreover, in the SA search processes, we have that, given a tree and a valid tree operation, the probability of accepting the new solution is $\min\{e^{\Delta v/T},1\}$, where Δv is the possible change in the likelihood function after performing the operation, and *T* is the current temperature. The cooling process follows a geometric decay with a factor (cooling rate) *cr* of $10^{-2}$, i.e. the temperature at the *i*-th iteration is equal to $T_{i}=(1-10^{-2})T_{i-1}$ and $T_{0}=10^{4}$. The SA process stops when the temperature drops below a lower bound set at $10^{-3}$.

Since mutation losses are not as frequent as mutation gains, our approach allows to set an upper bound on *d*: the total number of deletions of the resulting tree. For example, in a Dollo-*k* model, we can consider only trees where each mutation is lost at most *k* times, but there are at most *d* nodes associated to mutation losses.

### 2.4 Visualization

Alongside the main tool, we produced a post-processing tool, called SASC-viz, that can be used to perform processing and filtering operations after the computation of the main tool. Notice that the following operations do not change the actual evolutionary history computed but only how it is displayed.

- Collapsing simple paths: when this option is activated, all simple non-branching paths are collapsed, i.e. if a node has only one child, then such node is merged with its child;
- Collapsing low-support paths: when this option is activated, if the support of a node *i* is lower than a specified value, then *i* is merged with its parent ρ(i), where the support *s_i_* of a mutation *i* is computed on the output tree as follows: let *st*(*i*) be the set of nodes in the subtree rooted at *i*, and let *C*(*i*) be the number of cells assigned to the node *i*. Then, the mutation support *s_i_* is:
-
$s_{i}=\frac{\sum_{u\in st(i)}C(u)}{\sum_{v\in st(\pi (i))}C(v)-C(\rho (i))}.$

We used SASC-viz to produce the pictures of the real dataset analyzed. In particular, Figures 6 and 7 are obtained by activating the collapsing simple paths option. Figure 5 was produced with more enabled options: by collapsing simple paths and collapsing low-support paths with threshold 5%.

## 3 Results

### 3.1 Results on simulated data

We have tested our method on simulated data, where the ground truth phylogeny is known. We recall that it is possible, however, that a completely different tree achieves a better likelihood on the input data than the one obtained via simulation. This problem is essentially unavoidable, since generating a progression that is the unique solution for the corresponding SCS input matrix would require the contrived addition of artifacts to both the desired tree and the input matrix. These artifacts would likely be so artificial that the resulting instance would not satisfy even the basic assumptions on cancer progression.

#### 3.1.1 Generating simulated datasets

To test the methods, we run three different experiments with increasingly sophisticated models, according to the parameter settings of Supplementary Table S1. In the first experiment, we explore a model with the possibility of mutation losses, a phenomenon, which has been evidenced by (Kuipers *et al.*, 2017); the second experiment is produced with a model using only different (mutation-specific) false-negative rates, as seen in the real data we use. For the third experiment, we combine the previous two to extend the simulation to the most general model in which mutations have the possibility to be lost, and each have a specific false-negative rate.

The false negative error rate distribution of the real datasets are obtained by analyzing the raw data of MGH36 and MGH64 from Tirosh *et al.* (2016) and comparing the mutation frequencies in the scRNA-seq data to the matching Whole-Exome Sequencing (WES) from bulk RNA sequencing, to deduce the drop-out frequency. We have analyzed more than 2000 mutations and observed that the distribution of the real data is consistent with a Beta distribution, which we have used as a base for the generation of our simulated data.

The values of different false-negative rates are randomly chosen from a *Beta distribution* B(α,β) with parameters α,β<1, to better simulate the values found in real datasets; the different values of the prior probabilities of mutation losses are produced using a *Triangular distribution* with lower limit *a*, upper limit *b* and mean *c*, indicated as T(a,c,b). Such distribution is usually used when only the mode, upper and lower bounds are known in a population as proxy for a fair estimation of real-case scenarios. A detailed description of the method used to simulate the datasets is available in the Supplementary Material.

#### 3.1.2 Evaluating the simulated datasets

For each of the three experiments, we measure the accuracy of SASC with two scores based on standard cancer progression measures used in various studies (Jahn *et al.*, 2016; Malikic *et al.*, 2019a), i.e. *Ancestor–Descendant* and *Different-Lineage* accuracies; a novel parsimony-based score based on the difference between the number of flips, i.e. changes from 0 to 1 and from 1 to 0, estimated by some tool to correct the input; and the actual number of flips introduced by the simulation process to induce the noise. Lastly, we evaluate the trees using the edit distance measure of Karpov *et al.* (2018). A detailed explanation of all the measures is available in the Supplementary Material. Note that none of the above mentioned metrics explicitly measures the ability of tools to correctly infer ISA violations.

Additionally to the aforementioned measures, we provide two accuracy measures for the estimation of false negatives: (i) an accuracy of the estimation of the average false-negative rate in the simulations and (ii) the value of the average, over the 50 trees, of the mean squared error (MSE) over the set of estimations, for each mutation, of the mutation-specific false-negative rates. Note that (ii) gives an indication also of the *variance* of the estimation of false-negative rates, which is important when these rates are heterogeneous, and far from being normally distributed—something we see in real data that we use here and that is due to varying gene amplification and expression levels.

### 3.2 Results of the simulation experiments

We now detail the evaluation of the last experiment, which shows the most interesting results. Due to lack of space, we refer to the Supplementary Material for a detailed discussion on the other two, less general, experiments.

We decided to compare SASC against SCITE (Jahn *et al.*, 2016), SiFit (Zafar *et al.*, 2017) and SPhyR (El-Kebir, 2018). While B-SCITE (Malikic *et al.*, 2019a) is a clear improvement over SCITE, it combines single-cell data with bulk sequencing data—since we do not manage the latter kind of data, a fair comparison is not feasible. For the same reason, we do not compare against TRaIT (Ramazzotti *et al.*, 2019) and PhISCS (Malikic *et al.*, 2019a). OncoNEM (Ross and Markowetz, 2016) was excluded because it is not able to complete the execution on datasets as large as the ones used in the simulations. Each of the tools is properly run with millions of iterations and multiple restarts; the complete settings are available in the Supplementary Material.

**Fig. 2.**
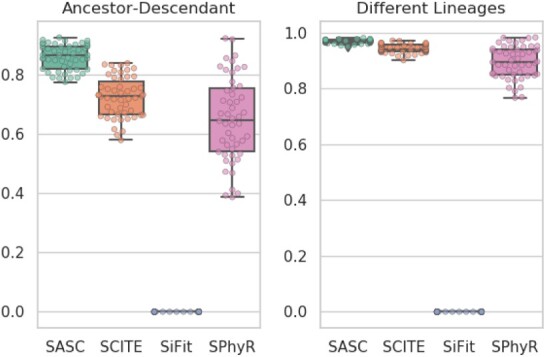
Accuracy results for the simulated experiment. In this experiment, SASC scores better than any other tool in these measures. Once again SiFit is the poorest scoring method. The accuracy of SPhyR lowers when mutation losses are included into the dataset and it is forced to employ a Dollo model. To the contrary, SASC performs the best when it utilizes the full extent of its capabilities, i.e. the handling of heterogeneous false-negative rates and mutation losses. Notice that larger values in both measures are better

**Fig. 3.**
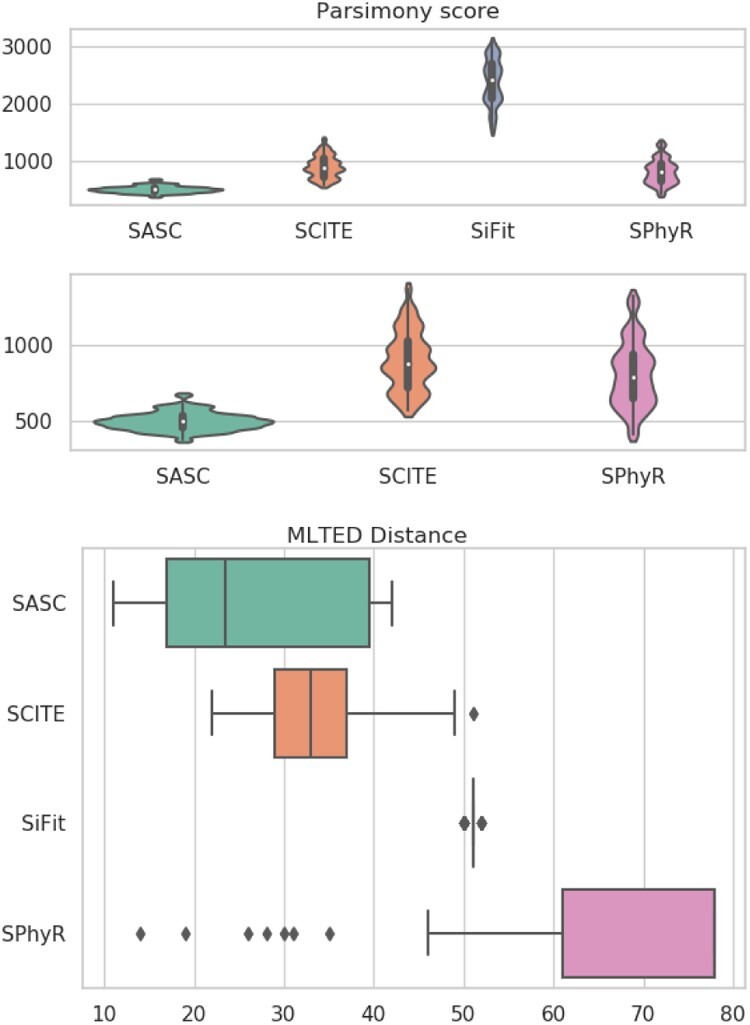
Accuracy results for the simulated experiment. According to these two measures, SASC scores better than any other tool. A clear performance drop is noticed when SPhyR is forced to employ a Dollo model. We represent the results of the parsimony score with and without SiFit, since its results are much different from the other ones. Notice that smaller values of both measures are better

**Fig. 4.**
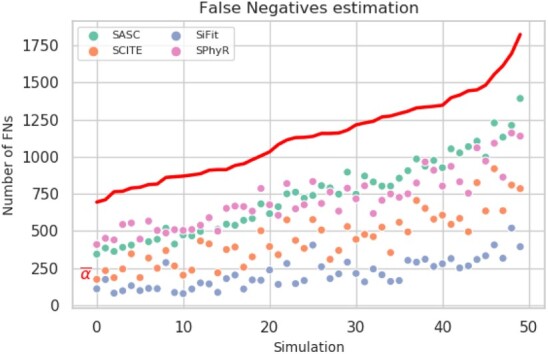
False-negative rates estimation for the simulated experiment. SASC estimates the false-negative rates better than the other tools, both in terms of average estimation, as well as MSE of the single rates for each mutation. Especially in the latter measure, we can notice a vast discrepancy in the accuracy of the estimation of false-negative rates. The thick red line is the average of the individual false-negative rates of the mutations in the ground truth

This experiment shows the results when the datasets contain both heterogeneous false negatives and deletions based on scRNA-seq error model, thus complementing the other experiments. SASC outperforms any other tool in every considered measure (Figs 2 and 3) and it also shows the best estimation of the false-negative rates in terms of average and MSE (Fig. 4). It is particularly interesting to notice the drop in performance of SPhyR when it is forced to employ the Dollo model, since this is the only experiment with mutation losses involved. It is also very clear that SASC outperforms all the available methods when it deals with heterogeneous false-negative rates and mutation losses. It also interesting to notice that SASC shows a much higher accuracy than the other two tools that allow mutational losses—SiFit and SPhyR—when such losses are present in the dataset.

### 3.3 Results on real cancer data

We test and compare SASC on four different datasets, comprising both scDNA-seq and scRNA-seq sequencing data. Due to lack of space, the figures of the trees inferred by the other methods are displayed in the Supplementary Material. Since SiFit exhibited poor performances on the simulated datasets, it is excluded in the comparison on real datasets.

#### 3.3.1 Oligodendroglioma IDH-mutated tumor

**Fig. 5.**
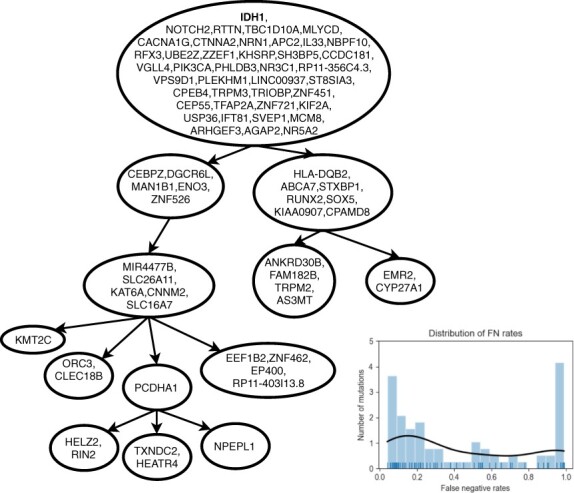
Tree inferred by SASC for the oligodendroglioma IDH-mutated MGH36 from Tirosh *et al.* (2016). The tree was computed using as input different false-negative rates for each mutation, whose distribution can be seen in the bottom-right corner plot. The picture was drawn using the SASC-viz post-processing tool

We test SASC on an oligodendroglioma IDH-mutated tumor; in particular, on cancer MGH36 (Tirosh *et al.*, 2016), consisting of 77 SNVs, distinguished from PCR false positives using matched WES, over 579 cells. Figure 5 shows the tree computed by SASC and the distribution of the false-negative rates (shown in the bottom-right corner plot). The distribution stresses the necessity of a method that considers heterogeneous false-negative rates, since there are two spikes of rates (at roughly 0.1 and 0.9), i.e. it is highly bimodal, and using the average of the rates would not be an accurate representation. In this particular tumor, no deletion was expected: this is confirmed by the inferred tree.

For the dataset MGH36 from Tirosh *et al.* (2016), there is no manually curated tree to compare the results of the tools, thus, we report the number of false negatives and false positives inferred by the methods, this is the number of flips from 0 to 1 and from 1 to 0, respectively, from the input to the output. The rationale for this score is to report a parsimony score of the algorithms; a comparison of the likelihood values will not be fair, since SASC uses a different formula than the other tools. Such score is shown in Supplementary Table S5; SASC introduces the lowest number of false negatives to obtain the solution, albeit being very close to SCITE (Supplementary Fig. S11), while SPhyR (Supplementary Fig. S14) infers the highest number.

#### 3.3.2 Childhood acute lymphoblastic leukemia

Furthermore, we test SASC on Childhood Acute Lymphoblastic Leukemia data from Gawad *et al.* (2014). In particular, we focus on Patients 4 and 5 of this study, given their large amount of both cells and mutations, as well as their complexity. Data on Patient 4 consist of 78 somatic SNVs over 143 cells, while Patient 5 is affected by 104 somatic SNVs over 96 cells. The original study estimated an allelic drop-out rate of <30%. Since the trees in Gawad *et al.* (2014) determined using expectation–maximization on a multivariate Bernoulli distribution model, are manually curated and of high quality, we select them as the ground truth.

To ensure the absence of doublets, i.e. noise produced by error due to the fact that two cells are sequenced instead of a unique cell, we preprocess the input using the *Single-Cell Genotyper* (SCG) tool (Roth *et al.*, 2016). SCG is a statistical model, which removes all cells of the datasets that are likely to be doublets.

**Fig. 6.**
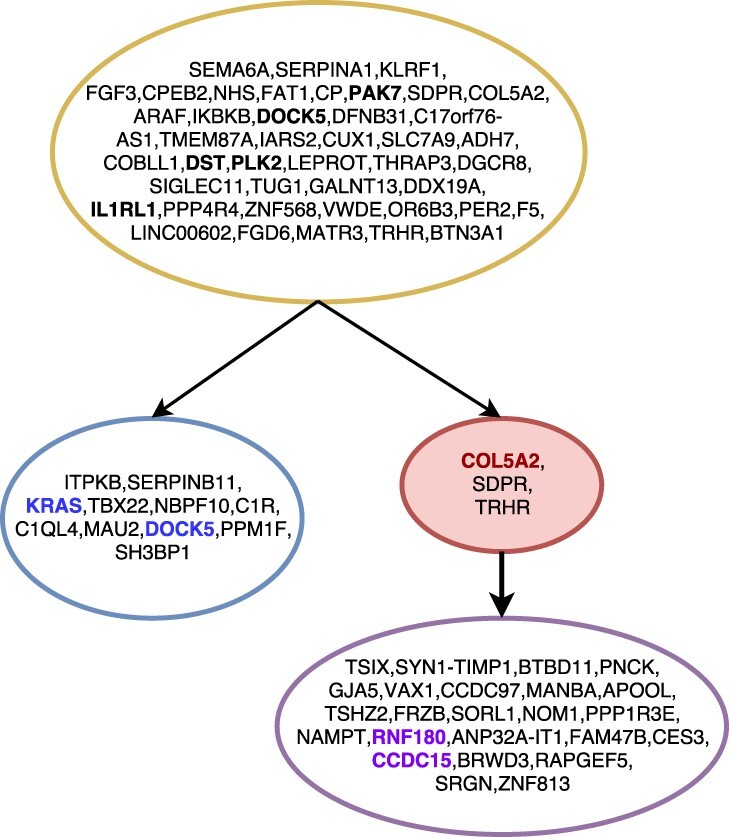
The tree inferred by SASC for Patient 4 of the Childhood Lymphoblastic Leukemia data from Gawad *et al.* (2014). Different clones are indicated with different colors. Red nodes indicate deletions of mutations, while bold-faced mutations are the mutations indicated as driver in the original sequencing study. Mutations in bold and colored are driver mutations for the clone with the same color. Mutations are clustered by collapsing simple linear paths. The picture was drawn using the SASC-viz post-processing tool

Figure 6 shows the tree inferred by SASC for Patient 4; SASC correctly infers the tree structure obtained in the study, as well as the size of the subclonal population. The driver mutations are correctly detected, and mutations COL5A2, SDPR and TRHR are inferred as deletions. Furthermore, bold-faced and colored mutations indicate the correctly placed specific driver mutations for the subclone of the same color. It is interesting to notice that, in the original study, the violet subclone does not have mutations COL5A2 and TRHR: these particular mutations are in fact deleted in the clone. This solution was found assuming a Dollo-1 phylogeny model with no restriction on the total number of deletions in the cancer progression.

**Fig. 7.**
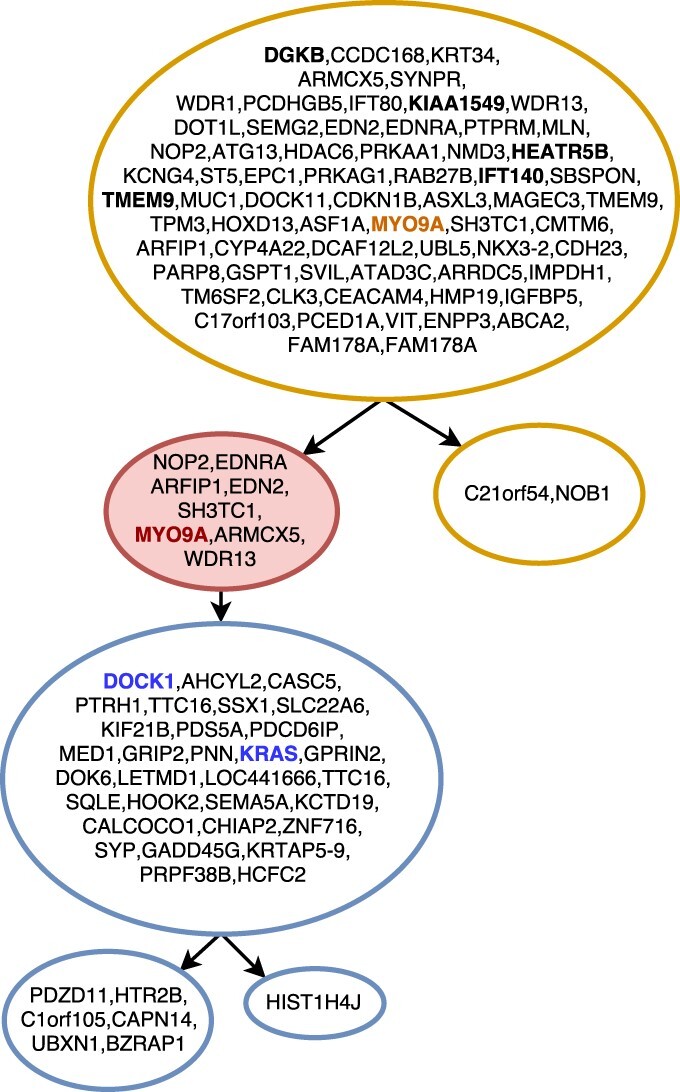
Tree inferred by SASC for Patient 5 of the Childhood Lymphoblastic Leukemia data from Gawad *et al.* (2014). Different clones are indicated with different colors, while the red-colored nodes indicate deletions of mutations, and mutations highlighted in bold are the mutations indicated as driver in the original sequencing study. Mutations bold-faced and colored are driver mutations for the same colored clone. Mutations are clustered by collapsing simple linear paths. The picture was drawn using the SASC-viz post-processing tool

In Figure 7, the inferred solution for Patient 5 of the same study is shown. As in the previous dataset, our inferred tree perfectly supports the hypotheses proposed in the original sequencing study: in fact, it correctly infers the topology of the tree, as well as the placement of driver mutations. Bold-faced mutations are the driver mutations for the tree or the subclone with the same color. This solution was found assuming a Dollo-1 phylogeny model with a restriction of 10 deletions in the cancer progression. As described in the Section 2.3, such values for *k* and *d* were empirically found to give the best likelihood.

Since the original study (Gawad *et al.*, 2014) provides manually curated trees, we can compare SASC, SCITE and SPHyR to them.

SCITE is run using the same setting used for SASC, i.e. the proposed values of false positive and false-negative rates. Supplementary Figure S9 shows the tree inferred by SCITE for Patient 4; the tree structure is similar to the one proposed in the article but it presents more clones. Furthermore, we highlighted in red all driver mutations that were not correctly detected, and in blue mutations that define a subclone and should be in the same subtree. Supplementary Figure S10 shows the tree inferred by SCITE for Patient 5 of the same study; the tree topology is correctly inferred, however mutations highlighted in red are driver mutations that were not correctly detected.

SPhyR is run using the same setting used for SASC, i.e. the proposed values of false positive and false-negative rates and assuming a Dollo-1 model. Supplementary Figure S12 shows the tree inferred by SPhyR for Patient 4; the tree structure is similar to the one proposed in the article. The drivers and distinct subclones are also correctly placed. Supplementary Figure S13 shows the tree inferred by SPhyR for Patient 5 of the same study; the tree topology is correctly inferred, however, it infers a large number of mutation losses, which is very unlikely and it is probably due to the fact that deletions are used, in this case, to correct false negatives at no cost in terms of likelihood function.

#### 3.3.3 Medulloblastoma

Lastly, we test the methods on Medulloblastoma patient BCH1031 from Hovestadt *et al.* (2019) consisting of 96 mutations over 330 cells. Given the complexity and the dimensions of the trees, we display them in the Supplementary Material. SASC and SPhyR computed the solution using a Dollo-2 phylogeny model.

Supplementary Figure S15 shows the tree inferred by SASC, which reported a total of two mutation losses. Both trees inferred by SASC and SCITE (Supplementary Fig. S16) express, as expected, various mutations correlated to the TUBB gene. On the other hand, SPhyR (Supplementary Fig. S17) inferred a total of 56 mutations over the 96 present in the sample. Furthermore, similarly to the previous experiment, SPhyR inferred a total of 24 mutational losses, which is very unlikely for so many losses to be present in a single sample, since evidence from Kuipers *et al.* (2017) suggests that this phenomenon is extremely rare. It is more likely that, also in this case, deletions are used to correct false negatives at no cost in terms of likelihood. Lastly, while SASC and SCITE each finished its computation in <2 h, SPhyR took more than 24 h.

## 4 Conclusion

We have presented SASC and we have shown that it is an accurate tool for inferring intra-tumor progression and subclonal composition from both scDNA-seq and scRNA-seq data. SASC manages cases with mutation losses and is robust to various sources of noise in all data.

We have tested SASC on three simulated datasets, and we have shown that SASC is able to outperform all tools when there are mutation losses, while being competitive with SCITE and SPhyR when there are no mutation losses.

We have tested SASC on three real datasets. SASC has inferred a likely phylogeny tree structure, correctly identifying the driver mutations and the decomposition of the clones. Furthermore, it has solved those large datasets in adequate runtime.

A particularly interesting example is given by the inferred tree in Figure 7. The corresponding input dataset in this case contains more than 5000 conflicts between mutations—each conflict is a pair of mutations witnessing a violation of the ISA. With only a slight relaxation of the ISA—the Dollo-1 model—SASC is able to infer an accurate solution with a total of only eight deletions, while perfect phylogeny methods would require a large number of changes to the entries in the input just to produce a feasible solution.

A future extension could be the inclusion of coverage information from the reads, as in Monovar (Zafar *et al.*, 2016) and SciΦ (Singer *et al.*, 2018), since it will also have an impact on the false-negative rates. Another direction is toward even more general models, e.g. allowing each mutation to appear more than once in the tree. Also in this case, special attention must be paid to keeping the model sufficiently restricted so that computation time does not explode, and inferred trees are still relevant from a biological point of view.

The need for a model that allows mutation losses has been established in Kuipers *et al.* (2017), but no clear consensus on the model that is most suited to represent the true evolution of tumors has been reached so far, to the best of our knowledge. In our article, we introduce and follow a restricted version of the Dollo-*k* model, where the number of mutations in each site and the number of overall mutations is limited—even though our method can be used also in a more relaxed setting. Determining which of the possible models is going to be the basis for effective and efficient tumor phylogeny inference is something that needs to be explored in the future, but it will likely need the development of different methods, and a deeper understanding of the models.

All the data produced and the experimental settings are publicly available and reproducible at SASC’s repository https://github.com/sciccolella/sasc.

## Supplementary Material

btaa722_Supplementary_Data
