# Supplementary material for: Inferring cancer progression from Single-Cell Sequencing while allowing mutation losses
Source: Bioinformatics. 2020 Aug 17;37(3):326–33. doi: 10.1093/bioinformatics/btaa722 (PMC8058767; doi:10.1093/bioinformatics/btaa722)
Supplement: btaa722_Supplementary_Data [file btaa722_supplementary_data.pdf]

Supplementary Material — for: Inferring Cancer Progression from Single-cell Sequencing while Allowing Mutation Losses

Simone Ciccolella[1], Camir Ricketts[2,3], Mauricio Soto Gomez[1], Murray Patterson[1,5], Dana Silverbush[4], Paola Bonizzoni[1], Iman Hajirasouliha[3,*], and Gianluca Della Vedova[1]

[1]Department of Computer Systems and Communication, University of Milano-Bicocca, Milan, Italy
[2]Tri-I Computational Biology & Medicine Graduate Program, Weill Cornell Medicine of Cornell University, NY, USA
[3]Institute for Computational Biomedicine, Department of Physiology and Biophysics, Englander Institute for Precision Medicine, The Meyer Cancer Center, Weill Cornell Medicine of Cornell University, NY, USA
[4]Harvard Medical School, Massachusetts General Hospital, MA, USA
[5]Fairfield University, Fairfield, CT, USA
[*]to whom the correspondence should be addressed

S1 Learning error rates and mutation loss probability

Given the uncertainty of the provided rates for false negatives, false positives and loss probability it is important to propose a method capable of learning them during the process of finding the maximum likelihood phylogeny, since the rates are usually estimates and therefore not certain. For this reason during the SA phase when searching a neighbor, `SASC` has a $q\%$ probability of questioning the provided rates and proposing new values for α_j, β and γ_j, representing respectively the false negative rate for mutation j, the false positive rate and the prior probability of losing mutation j. The value of q can be set by the user and as default is set to 10%.

The new value of a rate is randomly chosen from a normal distribution having the input rate as mean and a user-specified value as standard deviation. For example, assuming σ_α to be the standard deviation of α, a new false negative rate $\widetilde{\alpha_j} \sim \mathcal{N}(\alpha_j, \sigma_\alpha)$ is chosen for each mutation i according to a uniform distribution; in case such value is negative the absolute value is taken. The same principle is applied to learn the false positive rate and the loss prior probability.

This feature is disabled by default but it can can be enabled if the user do not has a fair amount of certainty about what the values of α, β and γ_j should be.

Fig. S1 shows how T_0 and cr affect the running time and the solution accuracy, the previously mentioned parameters were then chosen as defaults for the algorithm, although one can set different values when running the tool.

S2 Generating simulated datasets

For each of the three experiments, we produced dataset consisting of 50 randomly-generated clonal trees, according to the following procedure for (randomly) generating a tree. Given the number S of subclones, we generate a random tree on S nodes by adding a new node as a child of a random pre-existing one. Each of the M mutations $q_1, \ldots, q_M$ is then, uniformly at random, assigned to one of the s_i subclones. We allow at most a fixed number d of deletions in each clonal tree, *i.e.*,

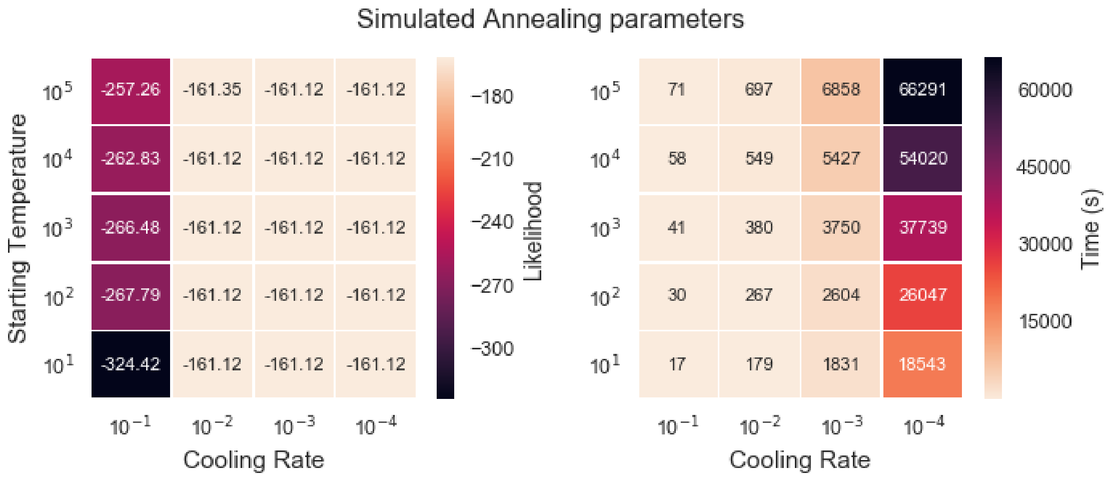

Figure S1: Variations of likelihood of solution (left) and running time (right) of SASC with respect to different values of starting temperatures and cooling rates of the Simulated Annealing process.

according to the prior probability $\gamma_j = P(L(j))$ of losing mutation q_j, at most d mutations are randomly selected to be lost. For each such loss, a new node q^- is created, and then inserted in the tree at a random valid position, *i.e.*, in the subtree where mutation q is gained but not in a path where it has already been lost.

To obtain the genotype profile of the N cells, we uniformly assign, at random, each cell to a node and derive its profile from the clonal tree. Finally to simulate noise in the data, we change a 0 entry to 1 with probability β to simulate false positives and a 1 entry to 0 with the corresponding probability α_j to simulate false negatives. Moreover, each entry has a probability μ to be a missing entry. All flips and missing values are independently distributed, without repetitions.

All the tools were run with the correct false positive rates, where the values of false negatives rates are heterogeneous, the average value of the α_j is given as input to the other tools, since SASC is the only method allowing for different false negative rates.

#	S	M	N	d	α	β	γ	μ
1	12	50	200	3	$\mathcal{B}(0.1, 0.1)$	3.4×10^{-5}	$\mathcal{T}(10^{-4}, 0.2, 1)$	0.15
S1	9	50	250	2	0.15	0.1	$\mathcal{T}(10^{-4}, 0.2, 1)$	0.2
S2	7	80	150	0	$\mathcal{B}(0.2, 0.2)$	3×10^{-5}	—	0.1

Table S1: **Parameters used to simulate the input matrices.** Here, S is the number of subclones, M is the number of mutations, N is the number of cells, d is the maximum number of allowed mutation deletions, α is the false negative rate, β the false positive rate, γ is the prior probability of a mutation loss and μ is the missing data rate.

S3 Evaluating the simulated datasets

Ancestor-Descendant accuracy: This measure considers all pairs of mutations (x, y) that are in an Ancestor-Descendant relationship in the ground truth tree T. For each such pair we check whether the ancestor-descendant relationship is conserved in the inferred tree I, in fact we calculate the number of mutations in an Ancestor-Descendant correctly inferred (true positives); the num-

ber of mutations that are incorrectly inferred to have an Ancestor-Descendant relationship (false positives); the number of mutations correctly inferred to not be Ancestor-Descendant (true negatives); finally the number of mutations incorrectly inferred to not have an Ancestor-Descendant relationship (false negatives). The score is defined by the F_1 score, that is the geometric mean of the precision and recall.

Different-Lineage accuracy: Just as the previous measure, we consider all pairs of mutations (x, y) that are not in an ancestor-descendant relationship, *i.e.* are in different branches of T. The score is defined, similarly to the previous measure, as the resulting F_1 score.

Parsimony Score: This is a parsimony-based measure. We measure the difference between the number of flips, *i.e.*, changes from 0 to 1 and from 1 to 0, estimated by some tool to correct the input, and the actual number of flips introduced by the simulation process to induce the noise. The rationale is that a good solution should be smaller, *i.e.*, closer to the correct amount of changes introduced by the simulation process. Formally, the Parsimony Score is defined as $|\mathcal{H}(S) - \mathcal{H}(E)|$ where $\mathcal{H}(S)$ is the total number of flips induced by the simulation, and $\mathcal{H}(E)$ is the number of flips estimated by the tool. While this measure does not consider the overall accuracy of a solution, it is a good estimation if used in conjunction with the previous ones.

MLTED [1]: Similar to the Parsimony score, we measure the *distance* between the tree inferred by some tool, and that of the ground truth — according to the recently presented *multi-labeled tree edit distance* (MLTED), which aims to define a distance tailored to cancer progression trees. Again the idea is that a good solution should be smaller to the ground truth, in terms of this distance. In [1], the MLTED is defined as the minimum number of label deletions, leaf deletions and vertex expansions to convert a pair of trees to the maximal common tree. The authors claim that such measure has been recently presented aiming to define a distance tailored to cancer progression trees, since most of the classic tree edit distances do not adapt well when applied to cancer phylogenies. Here we use the implementation of MLTED available at `https://github.com/khaled-rahman/MLTED` to compute the MLTED results reported below.

S4 Additional results on simulated data

S4.1 Experiment S1

The first experiment consists of the cases where only mutational losses occur, thus representing our simplest model, based on scDNA-seq error model. From Figures S2 , S3, S4 we see that SPhyR outperforms all other tools, while `SASC` and SCITE score almost identically. SiFit on the other hand shows poor results in all accounted measures. This is an expected result, since this are the experimental settings for which SPhyR was designed and, given its ILP nature, it is able to achieve a near-optimal solution in most cases.

S4.2 Experiment S2

In this experiment we want to focus at the cases where a heterogeneous set of false negatives is present in the data while no deletion is allowed, thus simulating errors from scRNA-seq data without any loss of mutation. From Fig. S5 we see a clear improvement of `SASC` over SCITE, while SPhyR shows an excellent accuracy. `SASC` and SPhyR perform very close in the Ancestor-Descendant, Different Lineages and Parsimony score while SCITE and SiFit show lower accuracy values. According to the MLTED distance (Fig. S6 `SASC` outperforms all the other methods, with a slight advantage over SPhyR. `SASC` also better infers the false negatives rate in both terms of average estimation and MSE, as seen in Fig. S7.

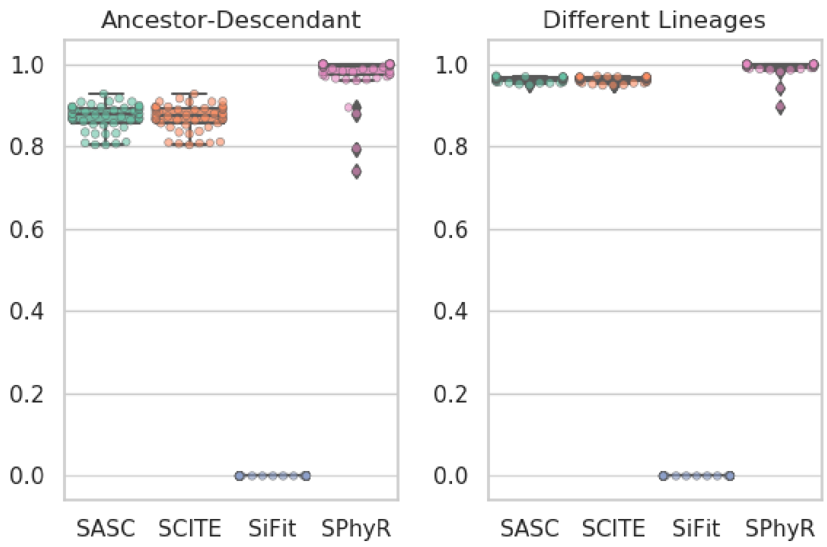

Figure S2: Accuracy results for Experiment 1, described in the section "Evaluating the simulated datasets". SPhyR outperforms all the other tools, since the experiment matches the settings it was designed for, and given given its ILP nature, it is able to achieve a near-optimal solution in most cases. `SASC` and SCITE score almost exactly, both with high accuracy, while SiFit shows lower results.

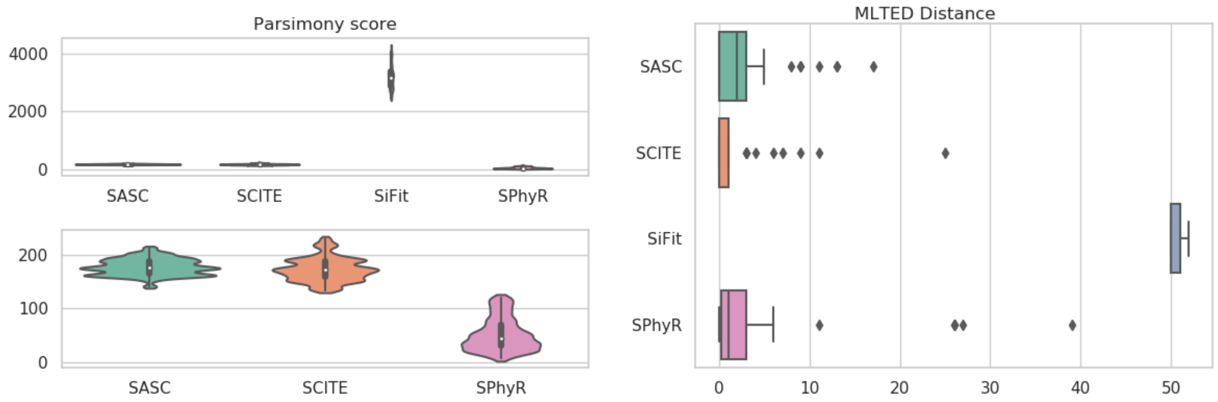

Figure S3: Parsimony scores and MLTED results for Experiment 1, described in the section "Evaluating the simulated datasets". SPhyR outperforms all the other tools, since the experiment matches the settings it was designed for, and given given its ILP nature, it is able to achieve a near-optimal solution in most cases. `SASC` and SCITE score almost exactly, both with high accuracy, while SiFit shows lower results.

S5 Setting used for running the tools

We made sure to run all the tools with appropriate amount of iterations and repetition to ensure the best possible result for each methods. The following tables shows the parameters of all `SASC`, SCITE, SPhyR and SiFit across the three experiments.

The number of iterations and repetitions was consistent across all the experiments; `SASC` was run with double the repetitions of SCITE and SiFit because as byproduct of the Simulated Annealing

Experiment 1

Method	Iterations	Repetitions	α	γ	k	d
`SASC`	—	10	0.15	γ	1	2
SCITE	2×10^6	5	0.15	—	—	—
SiFit	2×10^6	5	0.15	—	—	—
SPhyR	—	—	0.15	—	1	—

Table S2: Simulation setting for Experiment 1.

Experiment 2

Method	Iterations	Repetitions	α	γ	k	d
`SASC`	—	10	α	—	0	0
SCITE	2×10^6	5	average(α)	—	—	—
SiFit	2×10^6	5	average(α)	—	—	—
SPhyR	—	—	average(α)	—	0	—

Table S3: Simulation setting for Experiment 2.

Experiment 3

Method	Iterations	Repetitions	α	γ	k	d
`SASC`	—	10	0.15	γ	3	3
SCITE	2×10^6	5	average(α)	—	—	—
SiFit	2×10^6	5	average(α)	—	—	—
SPhyR	—	—	average(α)	—	3	—

Table S4: Simulation setting for Experiment 3.

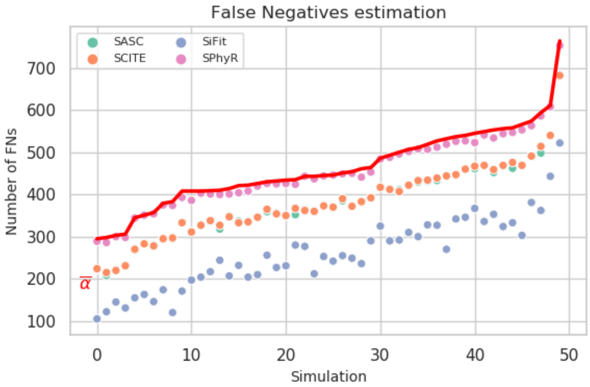

Mean Squared Error (average)

	SASC	SCITE	SiFit	SPhyR
MSE	7.62	7.64	5.20	9.07

Figure S4: False negative rates estimation for Experiment 1, described in the section "Evaluating the simulated datasets". SPhyR outperforms all the other tools, since the experiment matches the settings it was designed for, and given given its ILP nature, it is able to achieve a near-optimal solution in most cases. `SASC` and SCITE score almost exactly, both with high accuracy, while SiFit shows lower results.

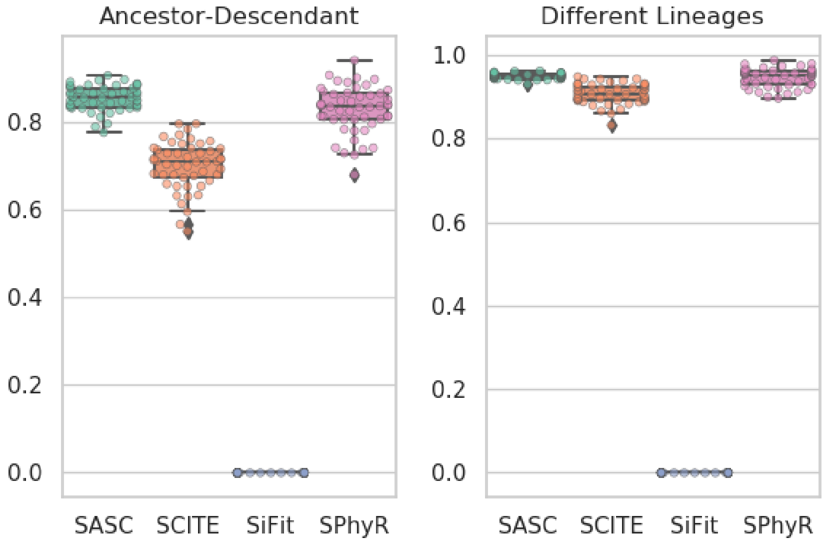

Figure S5: Accuracy results for Experiment 2, described in the section "Evaluating the simulated datasets". `SASC` slightly outperforms SCITE in both measures, while SiFit is shown to be the poorest scoring method. SPhyR scores slightly better than `SASC` on the Ancestor-Descendant accuracy and it outperforms all other tools on the Different Lineages measure. Notice that larger values of both measures are better.

algorithm the number of iterations performed is much smaller than what is set to the other tools. To compensate we doubled the number of repetitions. SPhyR does not require any number of iterations and repetitions given its ILP nature.

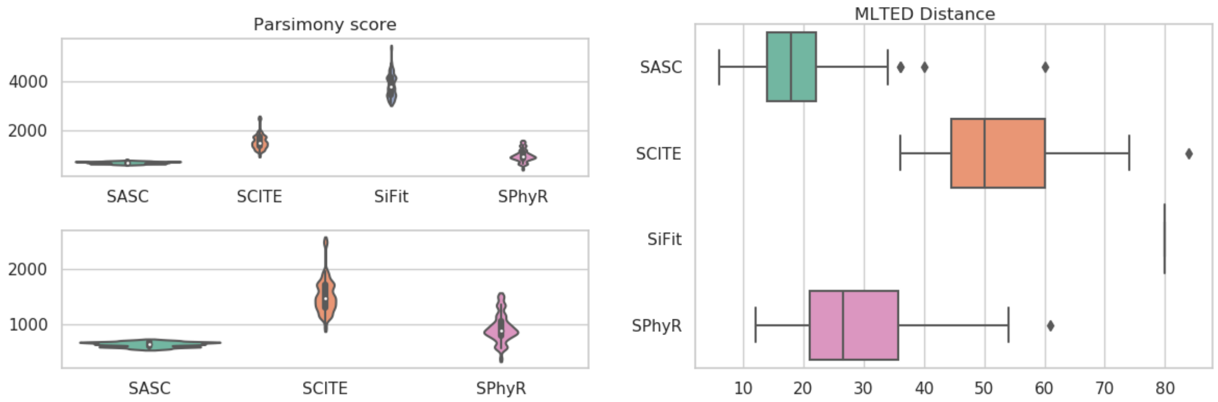

Figure S6: Parsimony scores and MLTED results for Experiment 2, described in the section "Evaluating the simulated datasets". `SASC` obtains better results than SCITE in both measure, while SPhyR's performance on the Parsimony score is very similar (albeit with a larger variance) to that of `SASC`. According to MLTED distance `SASC` scores better than any other tool, even though its performance is only slightly better than SPhyR. We represent the results of the parsimony score with and without SiFit, since its results are much different from the other ones. Notice that smaller values of both measures are better.

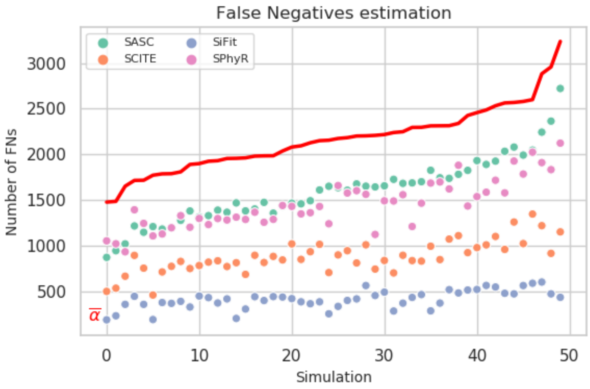

Mean Squared Error (average)

	SASC	SCITE	SiFit	SPhyR
MSE	7.60	7.64	5.20	9.07

Figure S7: False negative rates estimation for Experiment 2, described in the section "Evaluating the simulated datasets". `SASC` shows a more accurate estimation of the false negative rates than the other tools both in terms of average estimation as well as MSE of the single rates for each mutation. The thick red line is the average of the individual false negative rates of the mutations in the ground truth.

Across the tables α is the false negative rate and with an abuse of notation, it also means the distribution of false negative rates in Experiments 2 and 3; γ is the mutations loss rate distribution; k is the value of the Dollo-k phylogeny; d is the maximum number of deletion allowed in the tree.

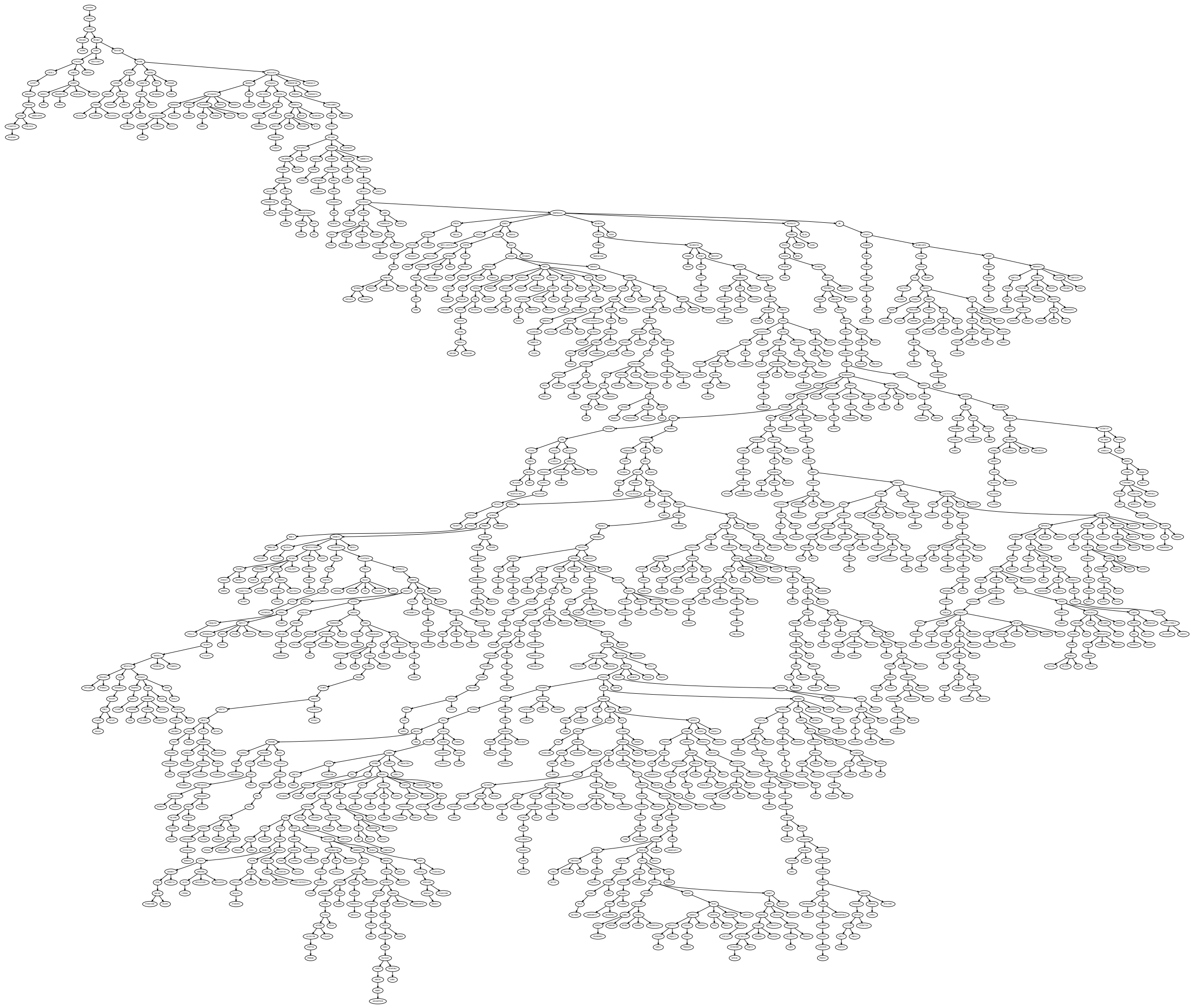

Figure S8: Tree inferred by SASC for patient MGH64 from [2] consisting of 1842 SNVs across 925. The computation was optimally completed in 4 hours using 16 cores and less than 200MB of RAM.

S6 Application of SASC to large dataset

We present here as example patient MGH64 from [2] consisting of 1842 SNVs across 925, which is the largest dataset we had access to in order to demonstrate the scalability of our method. The computation was optimally completed in 4 hours using 16 cores and less than 200MB of RAM. With such large datasets we suggest to increase the number of cores (parameter `-p`) used and the starting temperature (parameter `-S`).

	`SASC`	SCITE	SPhyR
FN	115	121	430
FP	7	6	10

Table S5: Number of false positive and false negative introduced on the MGH36 instance.

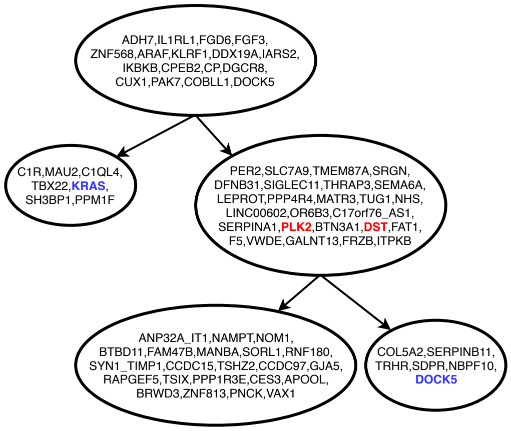

Figure S9: The tree inferred by SCITE for Patient 4 of Childhood Lymphoblastic Leukemia data from [3]. Mutations highlighted in red are driver mutations not correctly detected, while mutations highlighted in blue are two mutations that define a subclone and should be in the same cluster. Mutations are clustered by collapsing simple linear paths.

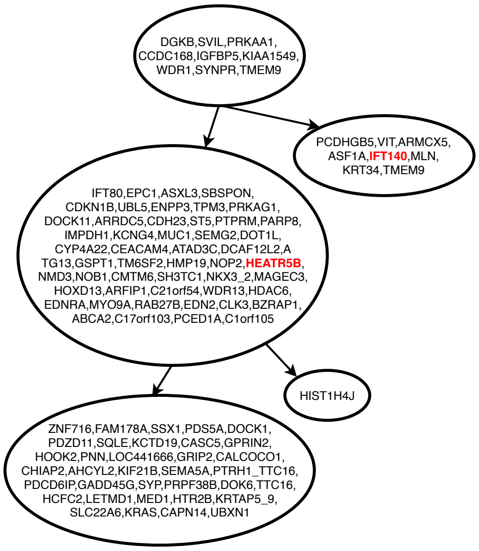

Figure S10: Tree inferred by SCITE for Patient 5 of Childhood Lymphoblastic Leukemia data from [3]. Mutations highlighted in red are driver mutations not correctly detected. Mutations are clustered by collapsing simple linear paths.

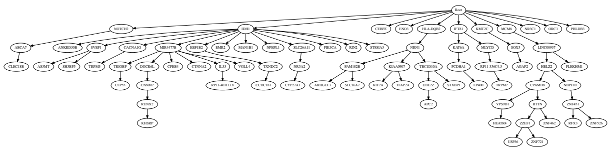

Figure S11: Tree inferred by SCITE for MGH36 of [2]. Nodes are not clustered.

References

[1] Nikolai Karpov, Salem Malikic, Md. Khaledur Rahman, and S. Cenk Sahinalp. A Multi-labeled Tree Edit Distance for Comparing "Clonal Trees" of Tumor Progression. In Laxmi Parida and

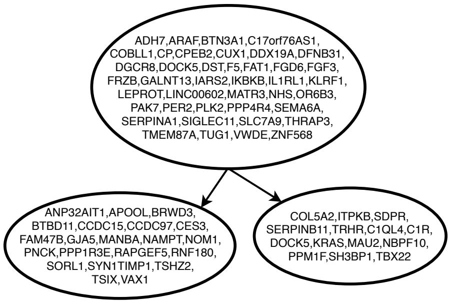

Figure S12: Tree inferred by SPhyR for Patient 4 of Childhood Lymphoblastic Leukemia data from [3]. Mutations are clustered by collapsing simple linear paths.

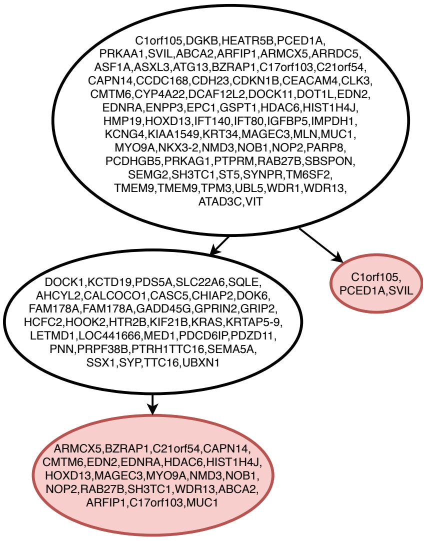

Figure S13: Tree inferred by SPhyR for Patient 5 of Childhood Lymphoblastic Leukemia data from [3]. Red nodes indicate deletions of mutations. Mutations are clustered by collapsing simple linear paths.

Esko Ukkonen, editors, *18th International Workshop on Algorithms in Bioinformatics (WABI 2018)*, volume 113 of *Leibniz International Proceedings in Informatics (LIPIcs)*, pages 22:1–22:19, Dagstuhl, Germany, 2018. Schloss Dagstuhl–Leibniz-Zentrum fuer Informatik.

[2] Itay Tirosh, Andrew S. Venteicher, Christine Hebert, Leah E. Escalante, Anoop P. Patel, Keren Yizhak, Jonathan M. Fisher, Christopher Rodman, Christopher Mount, Mariella G. Filbin, Cyril Neftel, Niyati Desai, Jackson Nyman, Benjamin Izar, Christina C. Luo, Joshua M. Francis, Aanand A. Patel, Maristela L. Onozato, Nicolo Riggi, Kenneth J. Livak, Dave Gennert, Rahul Satija, Brian V. Nahed, William T. Curry, Robert L. Martuza, Ravindra Mylvaganam, A. John Iafrate, Matthew P. Frosch, Todd R. Golub, Miguel N. Rivera, Gad Getz, Orit Rozenblatt-Rosen, Daniel P. Cahill, Michelle Monje, Bradley E. Bernstein, David N. Louis, Aviv Regev, and Mario L. Suvà. Single-cell rna-seq supports a developmental hierarchy in human oligodendroglioma. *Nature*, 539:309 EP –, Nov 2016.

[3] Charles Gawad, Winston Koh, and Stephen R. Quake. Dissecting the clonal origins of childhood acute lymphoblastic leukemia by single-cell genomics. *Proceedings of the National Academy of*

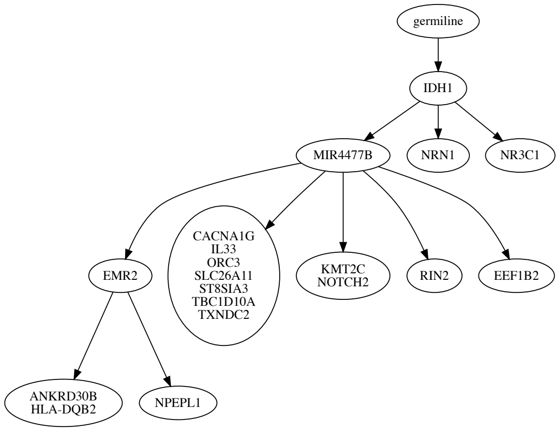

Figure S14: Tree inferred by SPhyR for MGH36 of [2]. A total of 20 mutations are reported by SPhyR out of the 77 present in the dataset; nodes are clustered by SPhyR.

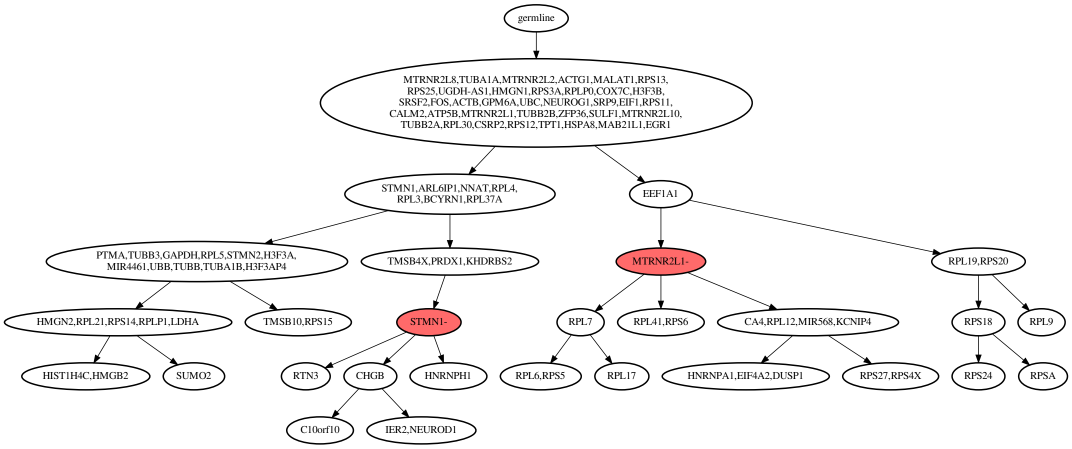

Figure S15: The tree inferred by `SASC` for BCH1031 of Medulloblastoma data from [4]. Red nodes indicate deletions of mutations. Mutations are clustered by collapsing support lower than 20%. The picture was drawn using the `SASC-viz` post-processing tool.

Sciences, 111(50):17947–17952, 2014.

[4] Volker Hovestadt, Kyle S. Smith, Laure Bihannic, Mariella G. Filbin, McKenzie L. Shaw, Alicia Baumgartner, John C. DeWitt, Andrew Groves, Lisa Mayr, Hannah R. Weisman, Alyssa R. Richman, Marni E. Shore, Liliana Goumnerova, Celeste Rosencrance, Robert A. Carter, Timothy N. Phoenix, Jennifer L. Hadley, Yiai Tong, Jim Houston, Richard A. Ashmun, Michael DeCuypere, Tanvi Sharma, Diane Flasch, Antonina Silkov, Keith L. Ligon, Scott L. Pomeroy, Miguel N. Rivera, Orit Rozenblatt-Rosen, Jessica M. Rusert, Robert J. Wechsler-Reya, Xiao-Nan Li, Andreas Peyrl, Johannes Gojo, Dominik Kirchhofer, Daniela Lötsch, Thomas Czech, Christian Dorfer, Christine Haberler, Rene Geyeregger, Angela Halfmann, Charles Gawad, John Easton, Stefan M. Pfister, Aviv Regev, Amar Gajjar, Brent A. Orr, Irene Slavc, Giles W. Robin-

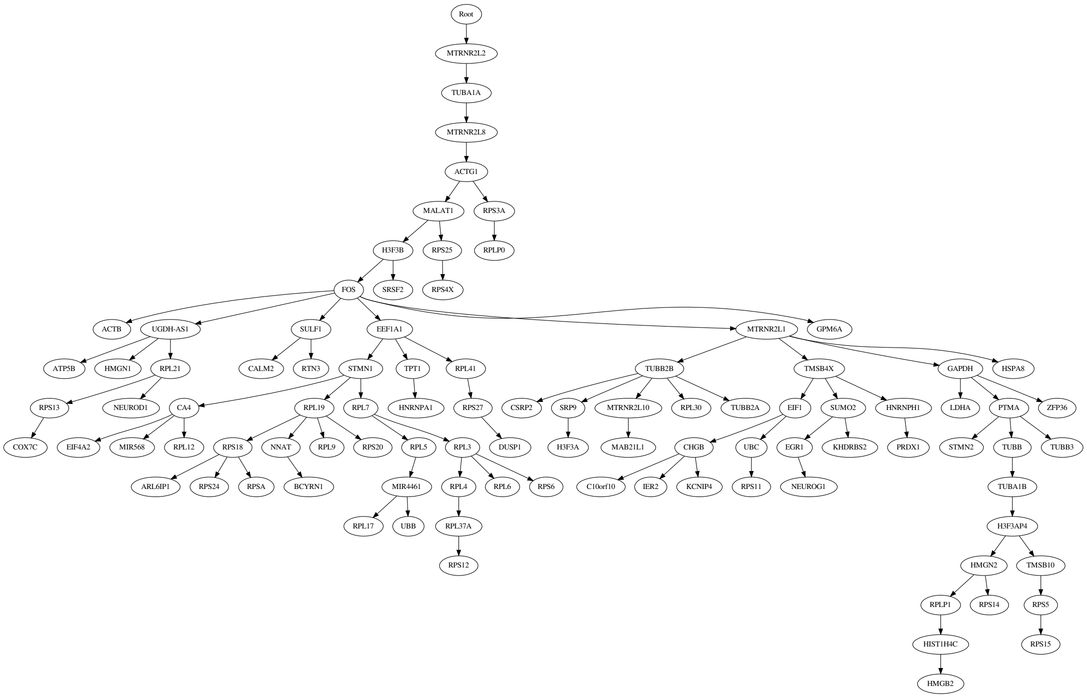

Figure S16: The tree inferred by SCITE for BCH1031 of Medulloblastoma data from [4].

son, Bradley E. Bernstein, Mario L. Suvà, and Paul A. Northcott. Resolving medulloblastoma cellular architecture by single-cell genomics. *Nature*, 572(7767):74–79, Aug 2019.

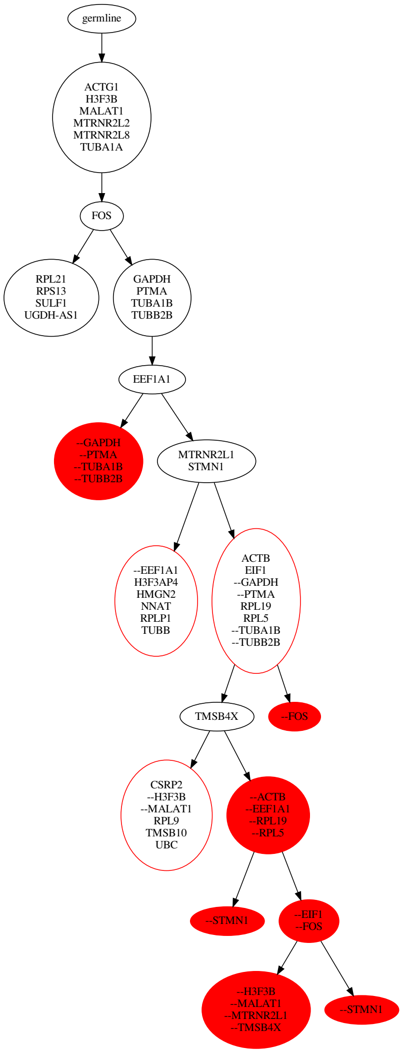

Figure S17: The tree inferred by SPhyR for BCH1031 of Medulloblastoma data from [4]. Fully red colored nodes represent nodes with all losses, while nodes with red edges contain both acquisition and losses. SPhyR inferred a total of 56 mutations over the 96 present in the sample. A total 24 losses are inferred in the tree.
